# Therapeutic effects of oral administration of lytic *Salmonella* phages in a mouse model of non-typhoidal salmonellosis

**DOI:** 10.3389/fmicb.2022.955136

**Published:** 2022-10-10

**Authors:** Chutikarn Sukjoi, Songphon Buddhasiri, Arishabhas Tantibhadrasapa, Thattawan Kaewsakhorn, Preeda Phothaworn, Janet Y. Nale, Angela V. Lopez-Garcia, Manal AbuOun, Muna F. Anjum, Danish J. Malik, Edouard E. Galyov, Martha R. J. Clokie, Sunee Korbsrisate, Parameth Thiennimitr

**Affiliations:** ^1^Department of Microbiology, Faculty of Medicine, Chiang Mai University, Chiang Mai, Thailand; ^2^Department of Veterinary Biosciences and Veterinary Public Health, Faculty of Veterinary Medicine, Chiang Mai University, Chiang Mai, Thailand; ^3^Department of Immunology, Faculty of Medicine Siriraj Hospital, Mahidol University, Bangkok, Thailand; ^4^Department of Veterinary and Animal Science, Northern Faculty, Scotland’s Rural College, Inverness, United Kingdom; ^5^Department of Bacteriology, Animal and Plant Health Agency, Weybridge, United Kingdom; ^6^Department of Chemical Engineering, Loughborough University, Loughborough, United Kingdom; ^7^Department of Genetics and Genome Biology, University of Leicester, Leicester, United Kingdom; ^8^Research Center of Microbial Diversity and Sustainable Utilization, Chiang Mai University, Chiang Mai, Thailand; ^9^Center of Multidisciplinary Technology for Advanced Medicine, Faculty of Medicine, Chiang Mai University, Chiang Mai, Thailand

**Keywords:** bacteriophage therapy, *Salmonella enterica* Typhimurium, foodborne pathogen, acute non-typhoidal salmonellosis, mouse colitis model, inflammatory response

## Abstract

Acute non-typhoidal salmonellosis (NTS) caused by a Gram-negative bacterium *Salmonella enterica* serovar Typhimurium (*S.* Tm) is one of the most common bacterial foodborne diseases worldwide. Bacteriophages (phages) can specifically target and lyse their host bacteria, including the multidrug-resistant strains, without collateral damage to other bacteria in the community. However, the therapeutic use of *Salmonella* phages *in vivo* is still poorly investigated. *Salmonella* phages ST-W77 and SE-W109 have previously been shown by our group to be useful for biocontrol properties. Here, we tested whether phages ST-W77 and SE-W109 can reduce *Salmonella* invasion into cultured human cells and confer a therapeutic benefit for acute NTS in a mammalian host. Human colonocytes, T84 cells, were treated with phages ST-W77, SE-W109, and its combination for 5 min before *S.* Tm infection. Gentamicin protection assays demonstrated that ST-W77 and SE-W109 significantly reduced *S.* Tm invasion and inflammatory response in human colonocytes. Next, streptomycin-pretreated mice were orally infected with *S.* Tm (10^8^ CFU/mouse) and treated with a single or a combination of ST-W77 and SE-W109 (10^10^ PFU/mouse for 4 days) by oral feeding. Our data showed that phage-treated mice had lower *S.* Tm numbers and tissue inflammation compared to the untreated mice. Our study also revealed that ST-W77 and SE-W109 persist in the mouse gut lumen, but not in systemic sites. Together, these data suggested that *Salmonella* phages ST-W77 and SE-W109 could be further developed as an alternative approach for treating an acute NTS in mammalian hosts.

## Introduction

*Salmonella enterica* serovar Typhimurium (*S*. Tm) is a Gram-negative bacterium in the family Enterobacteriaceae. Human infection caused by *S.* Tm through the fecal-oral route transmission is called “acute non-typhoidal salmonellosis (NTS).” Common symptoms of an acute NTS are high-grade fever, abdominal pain, and diarrhea, which usually develop on days two or three after ingesting *S.* Tm contaminated food or drink. Although most acute NTS patients with proper supportive treatments would recover within a few days, some immunocompromised patients could develop an invasive NTS (iNTS), a life-threatening form of NTS (Morpeth et al., 2009; Mather et al., 2018; Appiah et al., 2021). Appropriate antibiotics are essential to decrease the morbidity and mortality rate in iNTS patients.

Intravenous antibiotics are essential to treat an iNTS patient. However, the rise of multidrug resistance (MDR) *S.* Tm worldwide, including in Southeast Asia, increased the challenge (Mather et al., 2018; Whistler et al., 2018). Clinical strains of *S.* Tm from Thailand and worldwide exhibit resistance to several groups of the commonly used antibacterial agents such as fluoroquinolones and cephalosporins (Patchanee et al., 2015, 2017; Hengkrawit and Tangjade, 2022). These concerns raise the urgent need to find alternative approaches to an antibiotic for acute NTS, such as probiotics or bacteriophages (Deriu et al., 2013; Nikkhahi et al., 2017).

Bacteriophages (phages) are a group of particular viruses that infect and kill their bacterial hosts. Phages are usually found in common environmental reservoirs where their hosts are found (Clokie et al., 2011). Most tailed phages belong to the family Myoviridae, Podoviridae, and Siphoviridae (Weinbauer, 2004). For several decades, phages have been considered an alternative treatment to MDR bacteria in human and animal infectious diseases (Salmond and Fineran, 2015; Harada et al., 2018; Jamal et al., 2019; Au et al., 2021). For example, a local application of a phage cocktail at a very low concentration gradually decreased *P. aeruginosa* burden in a human burn wound clinical trial (Jault et al., 2019). Recently, phage therapy improved the symptoms of disseminated cutaneous *Mycobacterium chelonae* infection in immunocompromised patient (Little et al., 2022). Moreover, the anti-*Salmonella* therapeutic effect of bacteriophages has been already demonstrated in agriculture, livestock, farm animals, and their related food products (Fiorentin et al., 2005; Svircev et al., 2018; Au et al., 2021; Thanki et al., 2022).

Our previous study in *Galleria mellonella*, a larvae model for several bacterial infections, demonstrated that the prophylactic regimen with a phage cocktail significantly increased the survival rate of *Salmonella*-infected larvae (Nale et al., 2020). Compared with co-infection and remedial regimens, the preventive phage regimen showed better protection and control against *Salmonella* infection in the haemolymph of the *Galleria*. We also found that *Salmonella* phages ST-W77 and SE-W109 isolated from sewages in Thailand conferred the highest broad host range activity to several serovars of *Salmonella enterica* (Phothaworn et al., 2020). ST-W77 belongs to Myovirus within the Viunalikevirus genus, and SE-W109 belongs to Siphovirus within the Jerseylikevirus genus. Both ST-W77 and SE-W109 phages conferred an obligate lytic lifestyle and lack of lysogeny-related genes in their genomes (Phothaworn et al., 2020). ST-W77 and SE-W109 can reduce *Salmonella* numbers in food products (milk and chicken meat) and hence have therapeutic potential for further development as a biocontrol agent in food (Phothaworn et al., 2020).

According to our previous studies (Nale et al., 2020; Phothaworn et al., 2020), the application of phages ST-W77 and SE-W109 as potential candidates for alternative treatment of MDR *Salmonella* infection in mammalian hosts should be explored. It had been reported that therapeutic administration of *Salmonella* phage increased the survival rate up to 60% in mice (Tang et al., 2019). Preadministration of a lytic phage reduced gut dysbiosis and intestinal inflammation in *S.* Tm-infected mice (Bao et al., 2022). However, the therapeutic effect of phages ST-W77 and SE-W109 in a mammalian host infected with *S.* Tm is still unknown.

In this study, we performed a gentamicin protection assay on human colonocytes, T84 cells, challenge with *S.* Tm and phages to investigate the efficacy of phages ST-W77 and SE-W109 in the prevention of *S.* Tm invasion. Then, we further investigated bacterial proliferation, gut and systemic tissue inflammation in *S.* Tm-infected mice treated with a single oral administration of phages ST-W77, SE-W109, and its combination. A viable bacterial count, qualitative polymerase chain reaction (qPCR), and histopathological study were used to determine the degree of *S.* Tm proliferation and tissue inflammation in mice.

## Materials and methods

### Ethical statement

The animal experiment protocol was reviewed and approved by the Animal Care and Use Committee, Chiang Mai University, Thailand, in accordance with the Association for Assessment and Accreditation of Laboratory Animal Care (AAALAC) guidelines (Approval no.2563/MC-0002). This work was approved by the Institutional Biosafety Committee, Faculty of Medicine, Chiang Mai University (Approval no. CMUIBC 02033/2562).

### Bacterial strain and cell culture

*Salmonella enterica* serovar Typhimurium strain IR715 (ATCC 14028 derivative with nalidixic acid resistance; *S.* Tm) was used (Stojiljkovic et al., 1995). *S.* Tm was grown at 37°C aerobically with shaking in Luria-Bertani (LB) broth (10 g/l tryptone, 5 g/l yeast extract, and 10 g/l NaCl; Difco, United States) for 16–18 h. A nalidixic acid (0.05 mg/ml; AppliChem, Germany) was used as a selective antibiotic for *S.* Tm in liquid and solid media. The list of bacterial and phage strains used in this study shown in Supplementary Table S1.

The human colonic epithelial cell T84 (ATCC CCL-248) purchased from the American Type Culture Collection (ATCC) was used for the invasion assay in this study. A completed media for the T84 was prepared by adding 5% fetal bovine serum (FBS; Hyclone, Singapore) and 1% Penicillin/Streptomycin (Gibco, United States) into Dulbecco’s Modified Eagle Medium (DMEM): F12 (Gibco, United States) contained 2.5 mM L-glutamine and 15 mM HEPES. Cells were grown at 37°C with 5% CO_2_ in a humidified condition.

### Phage preparation by spot assay

*Salmonella* phage ST-W77 and SE-W109 isolated from sewage samples in Thailand were prepared by a double-layer agar assay as previously described (Phothaworn et al., 2020). *S.* Tm lawn was formed by adding 100 μl of mid-log (exponential) phase liquid culture of *S.* Tm into 4 ml of 0.35% melted top LB agar supplemented with 10 mM CaCl_2_. Then, 10 μl of ten-fold serially diluted phage solution was spotted on the lawn, and a clear zone was observed at 18 h after the incubation at 37°C. Plaque-forming unit (PFU)/mL of phage solution was calculated by a ten-fold serially diluted phage solution.

### Invasion assay (gentamicin protection assay)

The invasion assay of *S.* Tm into human colonocytes was adapted from the previously described work (Winter et al., 2009). Briefly, 0.5 ml of T84 cells resuspended in the complete medium were seeded into 24-well plates at a density of ~10^5^ cells/well and incubated at 37°C with 5% CO_2_ for 24 h. The complete growth medium was replaced by the DMEM: F12 without FBS or antibiotics for an additional 24 h to synchronize the cells. Then, T84 cells were treated with 10 μl of phages 5 × 10^10^ PFU/mL in SM buffer into each well for 5 min before *S.* Tm IR715 adding at the multiplicity of infection (MOI) of 25 at 37°C for 1 h. The cells were subsequently washed with Dulbecco’s phosphate-buffered saline (DPBS; Hyclone, Singapore) and treated with 500 μl/well of 100 μg/ml gentamycin sulfate (AppliChem, Germany), and incubated at 37°C for 90 min. T84 cells were then washed with DPBS before adding 0.5 ml of 1% Triton-X-100 (Thermo Fisher Scientific, United States) in PBS to lyse the cells. The lysate was ten-fold serially diluted and enumerated on LB agar to determine the numbers of intracellular bacteria.

### Gene expressions in T84 cells by a qPCR

A qPCR from T84 cell culture was performed as previously described (Winter et al., 2009). In brief, 2 ml of T84 cells resuspended in the complete medium were seeded into 6-well plates at a density of ~10^6^ cells/well and incubated at 37°C with 5% CO_2_ for 24 h. The complete medium was replaced at 24 h prior to the assay by the DMEM: F12 without FBS and antibiotics. Then, T84 cells were treated by adding 10 μl of 1 × 10^11^ PFU/mL phages and *S.* Tm IR715 simultaneously at the MOI of 25 at 37°C for 3 h. Cells were harvested by adding 1 ml of Trizol (Ambion, United States) into each well. RNA from T84 cells was extracted by using TRIzol reagent (Thermo Fisher Scientific, United States) according to the manufacturer’s protocol. Then, complementary DNA (cDNA) was generated by using TaqMan reverse transcription reagents (Applied Biosystems, United States). A qPCR was performed using SYBR-Green based real-time PCR (Bioline, Tennessee, United States) in ViiA7 Real-Time PCR system (Applied Biosystems, United States) with primer pairs shown in Supplementary Table S2. Relative fold change of mRNA expressions was calculated by the comparative *Ct* method (2^−ΔΔ*CT*^). The expression levels of target mRNA genes (*IL-8, MIP-3A, and IL-1B*) were normalized with the housekeeping gene *GAPDH* as previously described (Winter et al., 2009).

### Mouse experiment

Female C57BL/6NJcl mice (*Mus musculus*) aged between 6 to 8-week-old were purchased from Nomura Siam International Company (Bangkok, Thailand). All mice were housed at the Laboratory Animal Center at Chiang Mai University in a temperature-controlled room (25°C) under a 12/12-h light/dark cycle with free access to food and water (*ad libitum*) for at least 1 week for acclimatization. Mice were divided into 4 groups (7 mice per group; Appiah et al., 2021) Saline-magnesium sulfate (SM) buffer-treated (Morpeth et al., 2009) ST-W77-treated, (Mather et al., 2018) SE-W109, and (Whistler et al., 2018) ST-W77 combined with SE-W109 (Supplementary Figure S1). All mice were orally fed with 100 μl of 200 mg/ml streptomycin sulfate (Lot number 5 K013479, AppliChem, Germany) 1 day prior to *S.* Tm infection. A streptomycin pre-treatment transiently impairs mouse gut colonization resistance. This treatment allows *S.* Tm to cause a gut inflammation similar to the pathology found in human acute NTS (Santos et al., 2001).

Streptomycin-pretreated mice were orally infected with 100 μl of 10^9^ CFU/ml *S.* Tm with an oral feeding. Then, all *S.* Tm-infected mice were orally fed with 100 μl of 10^11^ PFU/mL phage solution in SM buffer (10^10^ PFU/mouse) or SM buffer started at 1 h after *S.* Tm infection. Then, phage solutions were orally given to mice once daily for three consecutive days. The SM buffer was used as a control in the untreated group. Mouse feces were collected every day to enumerate the phage and *S.* Tm shedding. On day 4 post-infection (p.i.), all mice were euthanized, and mouse tissues were collected as previously described (Sarichai et al., 2020).

### Enumerations of *Salmonella* and phages from mouse fecal pellet and tissues

Mouse fecal pellets (2–3 pellets/mouse) were collected on day 1, 2, 3, and 4 p.i. Pellets were immediately weighed and homogenized using 1.0 mm diameter zirconium/silica beads (Biospec Products, United States) with a bead-beating machine. Then, ST-W77 and SE-W109 numbers (PFU/gm fecal pellet or tissue) were calculated by a double-layer agar assay.

Mouse tissues (colon content, cecal content, colon, cecum, ileum, liver, and spleen) were collected on day four p.i. when the most prominent gut inflammation occurred (Tsolis et al., 2011). Tissues were immediately weighed and homogenized using 1.0 mm diameter zirconium/silica beads (Biospec Products, Bartesville, United States) with a bead-beating machine. *S.* Tm colony-forming unit (CFU)/gm tissues were determined by a serial diluting technique on the LB agar with nalidixic acid (0.05 mg/ml).

### Gene expressions in mouse tissues by a qPCR

Mouse tissues (colon and spleen) were harvested at day 4 p.i. and immediately kept in RNA preservation (RNAstore Reagent, TIANGEN, China), then stored at -20°C until use according to the manufacturer’s instruction. Mouse colonic and splenic mRNA were isolated as previously described (Thiennimitr et al., 2011). A qPCR was performed using SYBR-Green-based real-time PCR (Bioline, Tennessee, United States) in ViiA7 Real-Time PCR system (Applied Biosystems, United States) with primer pairs shown in Supplementary Table S2. Relative fold change of mouse mRNA expressions (*Kc, IL-17, Zo-1, Ifn-g and Nos-2*) were calculated by the comparative Ct method using a mouse *Gapdh* as a housekeeping gene.

### Cecal histopathological study

Segments of mouse ceca were fixed in 10% buffered formalin, embedded in paraffin, and stained with hematoxylin and eosin (H&E; Sarichai et al., 2020). The slides were blindly scored by the veterinary pathologist using the criteria shown in Supplementary Table S3.

### Statistical analysis

Statistical analyses were performed using the GraphPad Prism (version 7) programs (GraphPad Software). Data were analyzed using the Student’s *t*-test to compare two groups and using one-way analysis of variance (ANOVA) when comparing three or more groups. The multiple comparison test by the GraphPad Prism 7.0 program assigned a *p*-value of <0.05, indicating a statistically significant difference. *, ** and *** indicate *p*-values <0.05, 0.01, and 0.001, respectively. Bars represent a geometric mean with the standard errors of the mean (SEM).

## Results

### Phage ST-W77 and SE-W109 reduced *Salmonella* invasiveness and inflammatory response in the human gut epithelial cells

**Figure 1:**
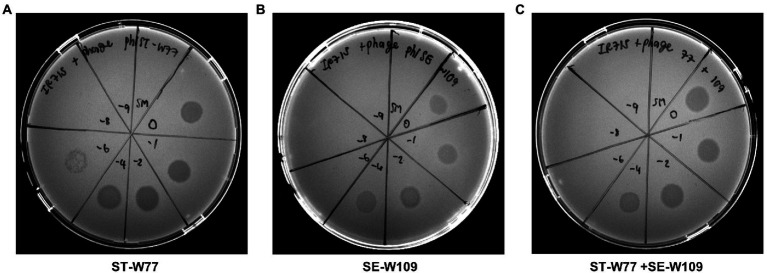
Susceptibility of *S.* Tm IR715 against phages ST-W77 and SE-W109. 10 μl of different phage dilutions in SM buffer (undiluted (10^−0^), 10^−2^, 10^−4^, 10^−6^, 10^−8^, and 10^−9^) were spotted on the *S.* Tm lawn, and clear zones were observed at 18 h after the incubation at 37°C. ST-W77 **(A)**, SE-W109 **(B)**, and a 1:1 combination of ST-W77 and SE-W109 **(C)**. SM buffer was used as a negative control.

Lytic activity of phage ST-W77 and SE-W109 on different serovars of non-typhoidal *Salmonella* strains was previously shown (Phothaworn et al., 2019). However, the lytic activity of ST-W77 and SE-W109 on *S.* Tm strain IR715 (the nalidixic acid derivatives of ATCC 14028) has never been reported. This study investigated whether ST-W77 and SE-W109 can lyse *S.* Tm IR715 *in vitro*. The spotting of 10 μl of each phage solution on the lawn of *S.* Tm IR715 resulted in clear zones for both phages compared to the SM buffer negative control (Figure 1).

**Figure 2:**
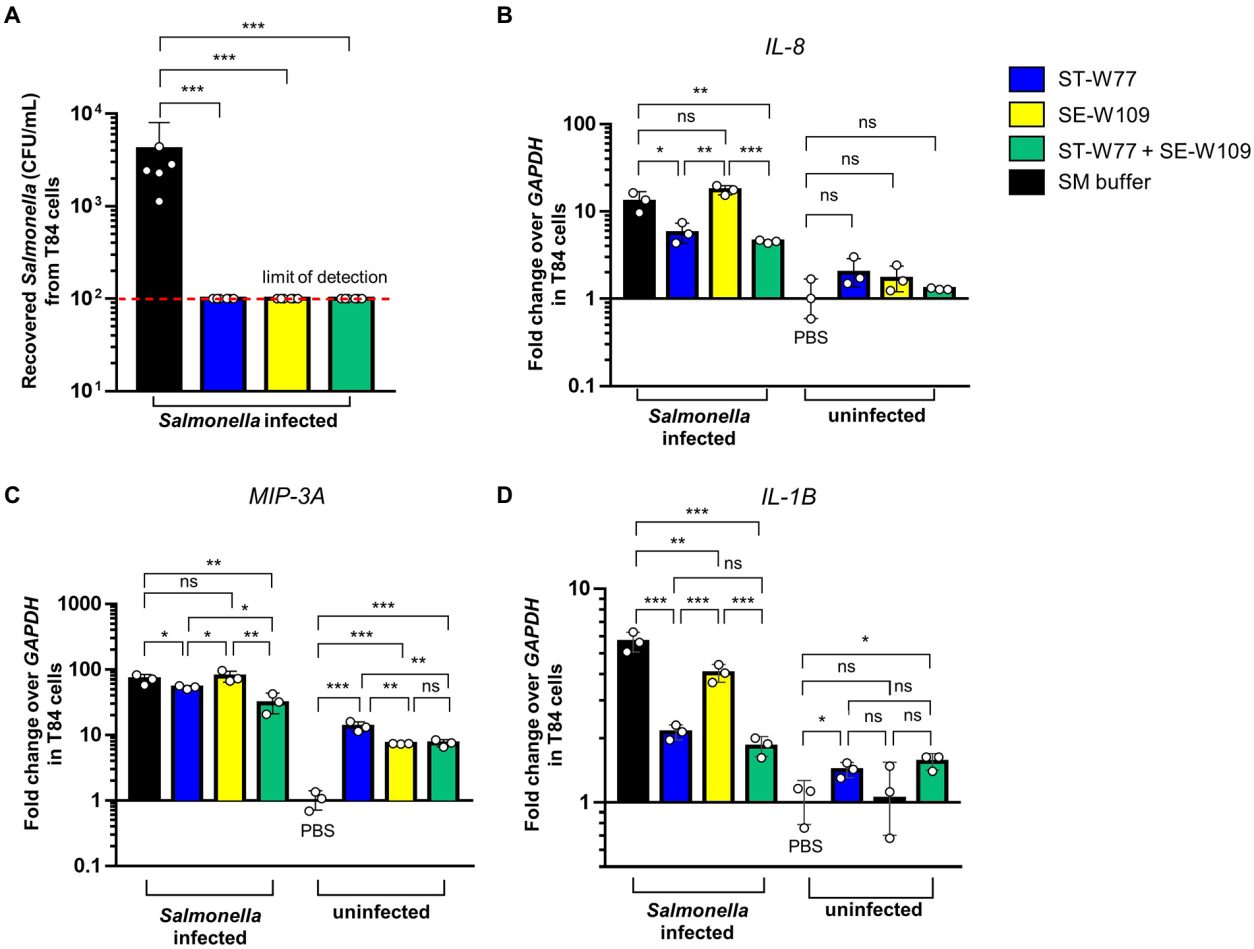
Efficiency of phages ST-W77 and SE-W109 in the protection against *S.* Tm invasion of human colonic epithelium. Human colonic epithelium (T84 cells) were seeded in a 24-well plate at a density of 10^5^ cells/well. Each well was added 10 μl of 5 × 10^10^ PFU/mL phages or SM buffer for 5 min before *S.* Typhimurium infection (MOI = 25). After the gentamicin protection for 90 min, cells were lysed and recovered bacterial numbers (CFU/mL) were counted **(A)**. The invasion assay was repeated in a 6-well-plate with the same MOI to observe proinflammatory gene expressions. Fold change of gene expressions of human *IL-8* **(B)**, *MIP-3A* **(C)**, and *IL-1B* **(D)** were determined by a qPCR using *GAPDH* as a housekeeping gene. Bars represent the geometric mean; error bars indicate geometric standard deviation. *, **, *** indicated *p* < 0.05, 0.01 and 0.001, respectively. ns, a non-statistically significant difference.

*S.* Tm is an invasive enteropathogen that uses several virulence factors to invade the host gut epithelium. Here, we investigated whether phages ST-W77 and SE-W109 can affect *S.* Tm invasiveness. The T84 cell, a human colonic epithelium, was used to assess *S.* Tm invasion using a gentamicin protection assay. Our data showed that both phages added to the cells concurrently with *S.* Tm significantly reduced the numbers (about 1.5 logs, *p*-value <0.001) of the invading *S.* Tm after 90 min of incubation (Figure 2A).

Next, we performed a qPCR to determine the relative fold change in mRNA expression of critical proinflammatory genes in T84 cells. We found that phage ST-W77 significantly decreased (*p*-value <0.05) the expression of two significant chemotactic cytokine genes (*IL-8* and *MIP-3A*; Figures 2B,C). Moreover, both phages reduced the fever-producing (pyrogenic) cytokine IL-1β gene *(IL-1B)* expression (Figure 2D). *IL-8*, encoding for interleukin (IL)-8 or chemokine (C-X-C motif) ligand 8 (CXCL8), is the important neutrophil chemotactic factor (Modi et al., 1990). *MIP-3A*, encoding for macrophage inflammatory protein-3 alpha (MIP-3A), is a macrophage proinflammatory chemokine in human (Rossi et al., 1997). IL-1β is a member of the IL-1 family that play a critical role in inflammation (Smirnova et al., 2002). However, phage SE-W109 reduced only *IL-1B* significantly (*p*-value <0.01; Figure 2D). The combination of ST-W77 and SE-W109 showed similar results to the single ST-W77 treatment (decreased *IL-8*, *MIP-3A*, and *IL-1B* expression).

Moreover, we tested whether each phage alone would activate an innate immune response in T84 cells without the *S.* Tm infection. We added 10 μl of 1 × 10^11^ PFU/mL phages in SM buffer into T84 cells resuspended in 6-well plates (10^6^ cells/well) without *S.* Tm challenge. Our data demonstrated that neither of the phages induce the expression of *IL-8* and *IL-1B* (Figures 2B,D). Interestingly, both phages increased the expression of *MIP-3A* by about ten folds in T84 cells without *S.* Tm infection (Figure 2C). Taken together, these data suggested that ST-W77 reduced proinflammatory response better than that of SE-W109 in human colonic epithelium infected with *S.* Tm. Moreover, expression of *MIP-3A* but not the *IL-8* and *IL-1B*, can increase in T84 cells after phage challenges without *S.* Tm infection. These suggested that phages ST-W77 and SE-W109 might some how activate expression of some human proinflammatory cytokines. However, the contamination of other bacterial components, especially a lipopolysaccharide (LPS or endotoxin), in a crude phage lysate might activate the host immune system (Boratynski and Szermer-Olearnik, 2017). Therefore, the removal of bacterial component contaminations are essential before phage applications.

### Phage ST-W77 and SE-W109 persist in mouse gut but not a systemic site

**Figure 3:**
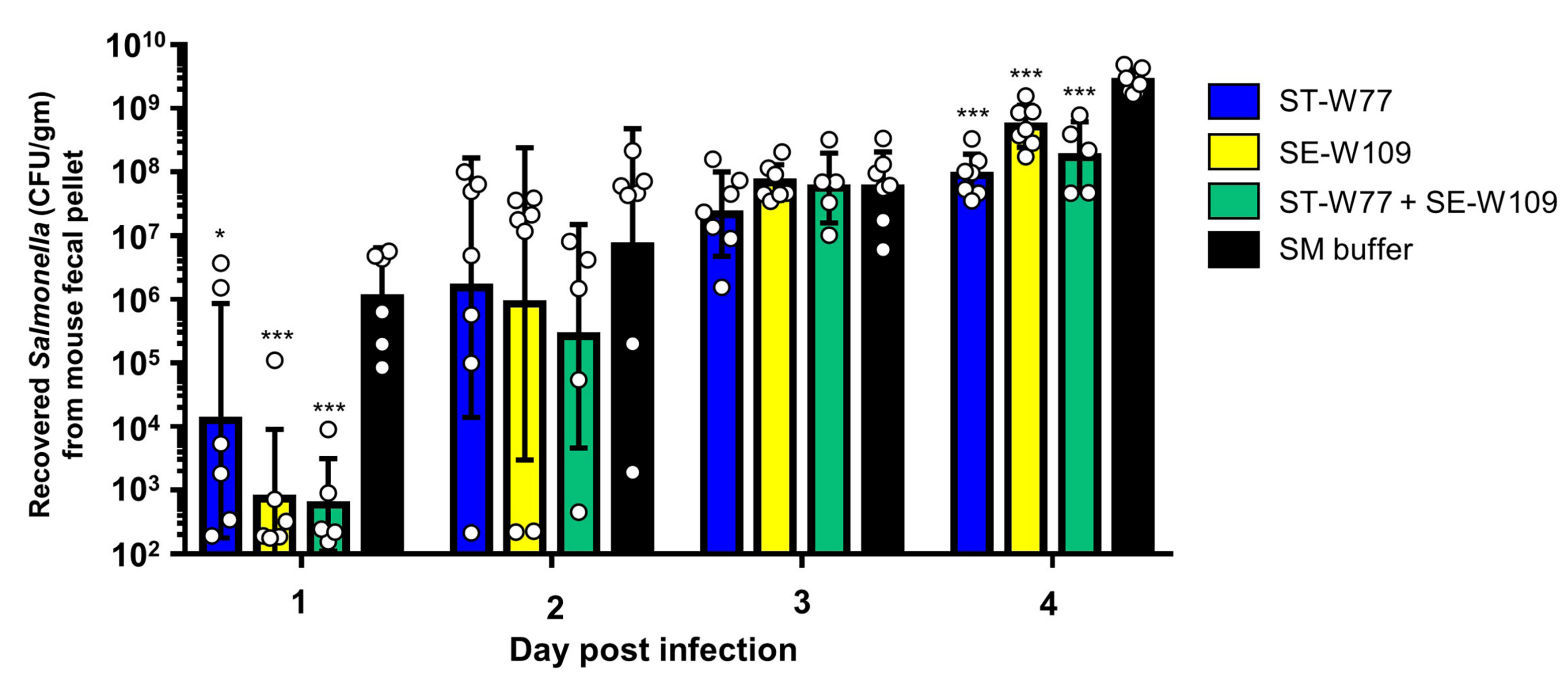
Oral feeding of phages (ST-W77 and SE-W109) reduced *Salmonella* shedding in mouse feces. The streptomycin-pretreated mouse colitis model of acute non-typhoidal salmonellosis was used. Orally treatment with phages (10^10^ PFU/mouse) was started 1 h after *S.* Tm infection and once daily for three consecutive days. On days 1, 2, 3, and 4 p.i., mouse fecal pellets from all groups, including ST-W77, SE-W109, and combination (ST-W77 + SE-W109), were collected for *S.* Tm CFU/gm. Bars represent the geometric mean; error bars indicate geometric standard deviation.* and *** indicated *p* < 0.01 and 0.001, respectively, compared to the SM buffer-treated group (black bar).

**Figure 4:**
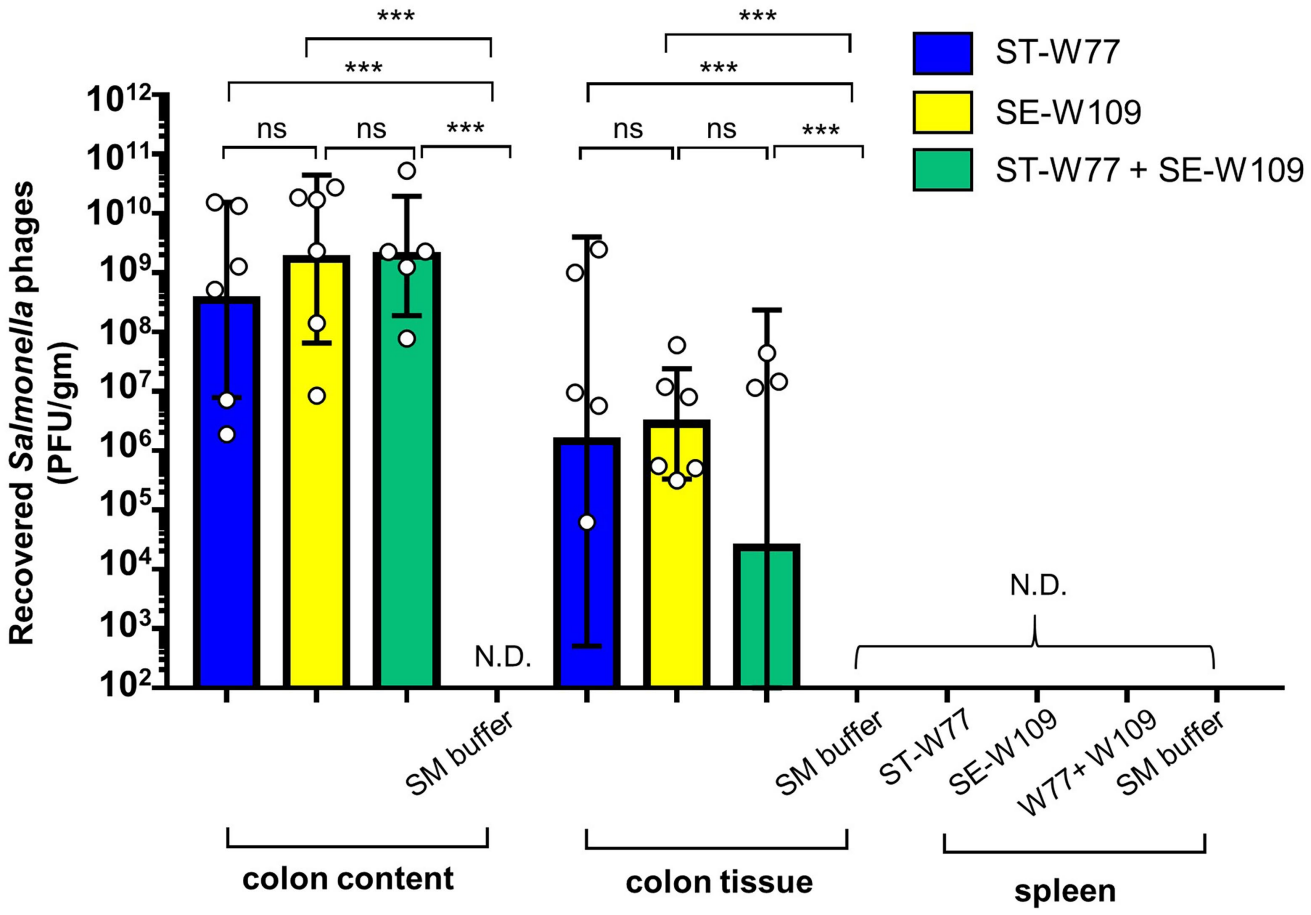
Phages ST-W77 and SE-W109 were recovered from mouse colon content, colon tissue but not spleen of *Salmonella*-infected mouse. All mice were orally fed with streptomycin sulfate 1 day prior to *S.* Tm infection (10^8^ CFU/mouse). Orally treatment with phages (10^10^ PFU/mouse) was started 1 h after *S.* Tm infection and once daily for three consecutive days. On day 4 p.i., mouse tissues were harvested for enumerated phage in each tissue (PFU/gm) by a double-layer plaque assay. Bars represent the geometric mean; error bars indicate geometric standard deviation. *** indicated *p* < 0.001 and ns, a non-statistically significant difference; N.D., not detectable.

To investigate the therapeutic effect of ST-W77 and SE-W109 *in vivo*, a streptomycin pre-treated mouse colitis model was used. The mice were orally infected with 10^8^ CFU of *S.* Tm IR715 and either treated with phages or with SM buffer in a control group. Mouse feces were collected on days 1, 2, 3, and 4 p.i. to determine the amount of shedding *Salmonella* (CFU/gm). Both phages significantly reduced *Salmonella* numbers in mouse feces compared to untreated mice on days 1 and 4 but not on days 2 and 3 p.i. (Figure 3). On day 4 p.i., all mice were euthanized, and recovered phages from mouse tissues were enumerated by plating (Figure 4). Our data suggest that both phages predominantly persisted in the mouse colon content (between 10^6^ to 10^11^ PFU/gm) and with the lower PFU (between 10^5^ to 10^9^ PFU/gm) in the mouse colon tissue. Phage numbers in the mouse tissues were similar for each of the two phages. No phage was detected from the mouse spleens in any of the groups. These data suggested that phages persist in the gut lumen and in colon tissue but not in systemic sites.

### Oral treatment with phage ST-W77 and SE-W109 significantly reduced *Salmonella* proliferation and inflammation in the mouse

**Figure 5:**
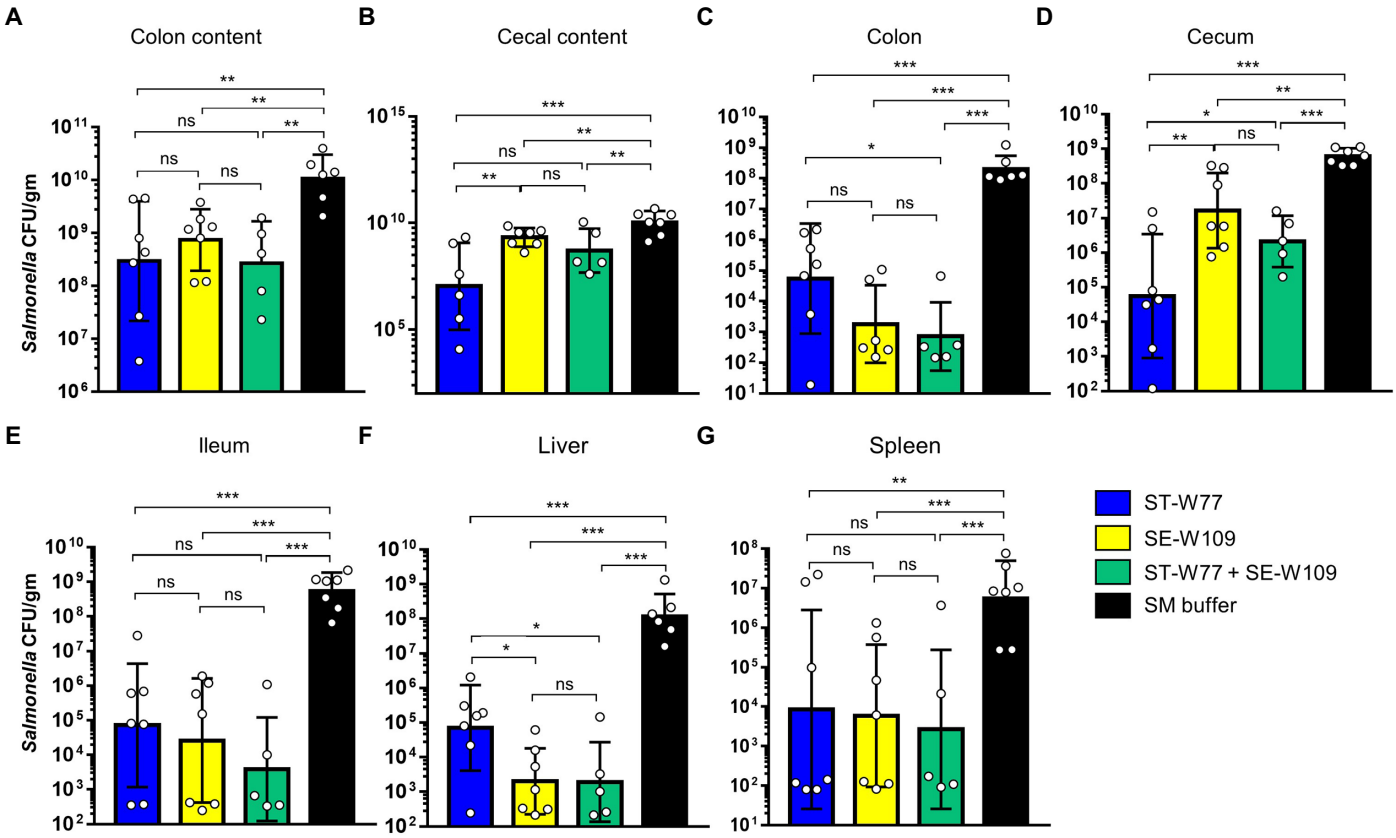
Both phages ST-W77 and SE-W109 significantly decreased *S.* Tm numbers in mouse gastrointestinal tract and systemic sites. All mice were orally fed with streptomycin sulfate 1 day prior to *S.* Tm infection (10^8^ CFU/mouse) and treated with phages (10^10^ PFU/mouse) for three consecutive days. On day 4 p.i., all mice were euthanized tissues were collected to detect *Salmonella* numbers in each tissue (CFU/gm) by a plating method. Numbers of *S.* Tm in the gut lumen **(A,B)**, local gut tissues **(C-E)**, and systemic tissues **(F,G)** were investigated. Bars represent the geometric mean; error bars indicate geometric standard deviation.*, **, *** indicated *p* < 0.05, 0.01 and 0.001, respectively. ns, a non-statistically significant difference.

After 4 days of oral treatment of *S.* Tm infected mice with phages (10^10^ PFU/mouse once daily), mice were euthanized, and tissues were collected and homogenized; the presence of bacteria was then assessed by plating (Figures 5A–G). Our data clearly demonstrate that infected mice treated with either of the phages had significantly fewer *S.* Tm in their tissues compared to the control SM buffer-treated mice. However, the treatment with ST-W77 caused more significant reduction in *S.* Tm numbers than treatment with SE-W109 alone or ST-W77 combined with SE-W109, in the cecum but not the colon. Treatment with either of the phages markedly reduced *S.* Tm numbers in the ileum (about 3–4 logs; *p*-value <0.001; Figure 5E). The systemic disseminating population of *S.* Tm in the liver and spleen was also significantly reduced in the phage-treated mice (Figures 5F,G). Interestingly, treatment with SE-W109 caused a greater reduction of *S.* Tm in the liver than treatment with ST-W77.

**Figure 6:**
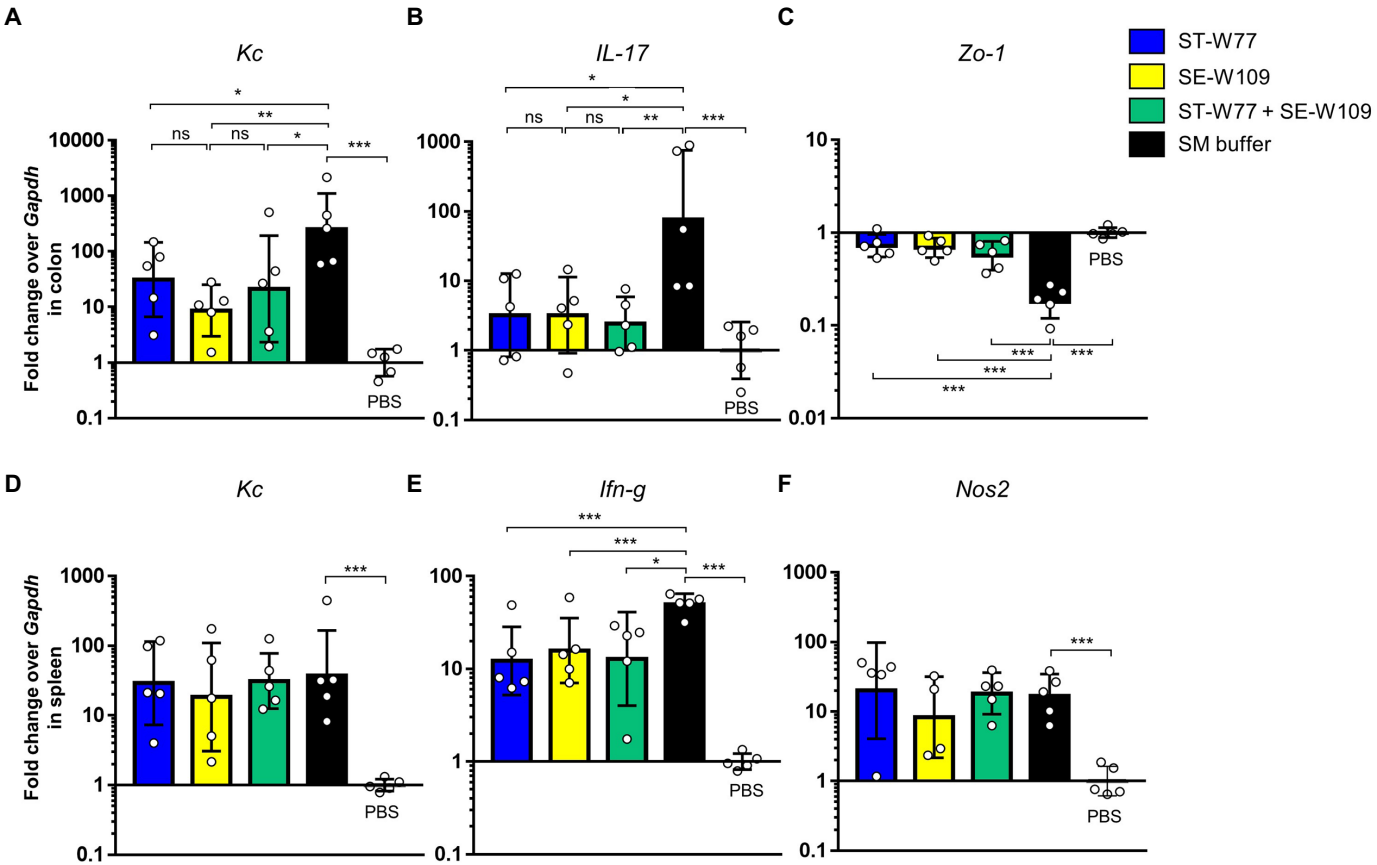
A reduction of proinflammatory makers in *S.* Tm infected mice orally treated with phages ST-W77 and SE-W109. Mouse tissues (colon and spleen) were collected for mRNA extraction and qPCR analysis. Colonic proinflammatory genes *Kc* **(A)**, *IL-17* **(B)**, and tight junction *Zo-1* **(C)** gene expressions were investigated. Expression of splenic proinflammatory genes *Kc* **(D)**, *Ifn-g* **(E)**, and *Nos2* **(F)** was shown. Bars represent the geometric mean; error bars indicate geometric standard deviation. *, **, *** indicated *p* < 0.05, 0.01 and 0.001, respectively. ns, non-statistically significant difference; PBS, phosphate-buffered saline.

Next, we determined therapeutic effect of phages on the inflammatory response caused by *S.* Tm infection in mice using qPCR assay and cecal histopathological study. ST-W77, SE-W109, and their combination conferred anti-inflammatory effects in both colon (Figures 6A–C) and spleen (Figures 6D–F). Our qPCR results indicated that the phages attenuated mouse gut inflammatory response. The lower expression of the colonic proinflammatory cytokine, keratinocyte chemoattractant (Kc), and IL-17 encoded by *Kc* and *IL-17* genes, respectively, were shown in phage-treated groups (Figures 6A,B) compared to the control group. In addition, increased colonic tight junction protein-1 or Zonula occludens-1 (Zo-1) expression encoded by the *Zo-1* gene (Figure 6C) was observed in both phage-treated groups.

**Figure 7:**
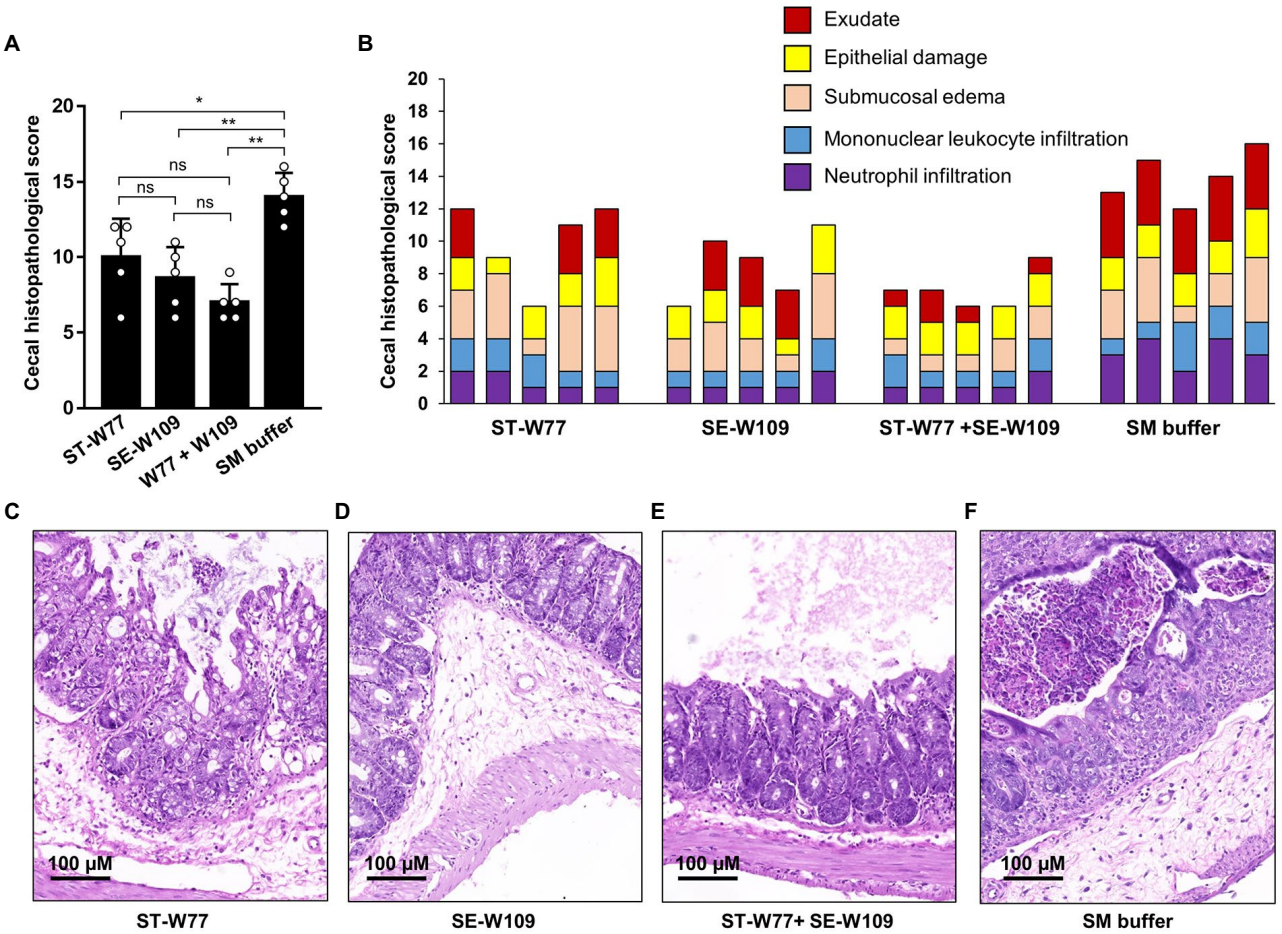
A significantly lower cecal histopathological score of *S.* Tm-infected mice orally treated with phages ST-W77 and SE-W109. Tips of mouse ceca were collected in 10% formalin and then hematoxylin–eosin (H&E) stained and double-blind scored by a veterinary pathologist using a histopathological score in Supplementary Table S3. The combined histopathological score **(A)** and the individual score with each criterion **(B)**. The representative picture of mouse cecum treated with ST-W77 **(C)**, SE-W109 **(D)**, combined ST-W77 and SE-W109 **(E)**, and SM buffer **(F)**. Bars represent the mean; error bars indicate standard deviation. * and **indicated *p* < 0.05 and 0.01, respectively. ns, a non-statistically significant difference.

To evaluate the effect of phages in mouse systemic inflammation, expression of three proinflammatory genes (*Kc*, *Ifn-g* and *Nos2*) in the spleen were determined. In both phage-treated groups reduced expression only *Ifn-g* but not *Kc* and *Nos2* (Figures 6D-F) was observed. Interferon (IFN)-γ, encoding by *Ifn-g*, is an important cytokine in a systemic phase of *Salmonella* infection (Santos, 2014). Expressions of *Kc* and *Nos2* (encoding for an inducible nitric oxide synthase or iNOS) were not different in the spleens of mice in all groups. We also evaluated the change in histopathology of mouse ceca by staining with haematoxylin and eosin (H & E). Our data showed that all phage-treated mice had significantly lower cecal histopathological scores compared to untreated mice (Figure 7). These data indicated that phage ST-W77 and SE-W109 attenuated inflammatory response caused by *S.* Tm infection in mice.

## Discussion

*S.* Tm is one of the major *Salmonella enterica* serovars that cause an acute gastroenteritis or acute NTS in human and farm animals such as swine, cattle and poultry (Santos et al., 2003, 2009). Considerable health problems and economic losses can occur after *S.* Tm contamination in the food supply chain. In addition, the increasing rates of MDR *S.* Tm raised concerns about the treatment of iNTS. The anti-*Salmonella* effect of two recently isolated obligate lytic *Salmonella* phages: ST-W77 and SE-W109, has been demonstrated in the previous studies *in vitro* (Nale et al., 2020; Phothaworn et al., 2020). Furthermore, a phage cocktail composed of ST-W77, SE-W109, and SPFM17 reduced the mortality rate of *Salmonella* infection in a *Galleria* model (Nale et al., 2020). To provide further justification and evidence-based support for the therapeutic usage of the two phages, we investigated the anti-*Salmonella* effect of ST-W77 and SE-W109 in a streptomycin-pretreated mouse colitis model of an acute NTS.

Phages ST-W77 and SE-W109 were able to lyse *S.* Tm IR715 strain and reduced its invasiveness to human colonic epithelium cells (Figures 1, 2, respectively). Reduced numbers of invading *S.* Tm has a potential significant advantage in the disease management as diminishing the bacterial invasion rate in the human gut, which is critical for the treatment of *S.* Tm infection. Our data indicated that the treatment with ST-W77 significantly reduced expression of three important proinflammatory cytokine genes (*IL-8*, *MIP-3A*, and *IL-1B*) better than SE-W109. This observation may be linked to the originally-isolated strain differences with ST-W77 specifically targeting the Typhimurium serovar., while SE-W109, the serovar Enteritidis. We observed that ST-W77 and SE-W109 did not induce the expression of *IL-8* and *IL-1B* but induced *MIP-3A* gene expression in the uninfected T84 cells (Figure 2C). Nonetheless, the observed activation of *MIP-3A* by ST-W77 and SE-W109 (about 10-fold increase) is still significantly lower than that in *S.* Tm-infected cells (about 100-fold increase). Another possibility of this observation might be explained by the contamination of bacterial components (e.g., DNA, protein or LPS) in the crude phage lysate (Boratynski and Szermer-Olearnik, 2017). In the absence of a *Salmonella* host, it is expected that phages should not activate a mammalian immune system. Nonetheless, other contributing factors such as microbial homeostasis, mucosal immunity, and immunological tolerance might play a role in phage-eukaryote interaction should be considered (Chatterjee and Duerkop, 2018). Taken together, our findings suggested that the purification of crude phage lysate before its application, especially for the phage therapy, to eliminate the contaminated bacterial components is essential. In addition, some phages may confer an immunomodulatory effect on mammalian cells even without their bacterial host and concurs with other reports (Gorski et al., 2012; Khatami et al., 2021; Safari et al., 2022).

Mammalian host immune response to both bacteria and phages could also determine the outcome of phage therapy (Hodyra-Stefaniak et al., 2015). In this study, both phages were not detected in the mouse spleen, indicating the gastrointestinal tract localized anti-*Salmonella* effect with possible low immunogenicity to mouse systemic immune response. To our knowledge, the effect of *Salmonella* lytic phage therapy on gut inflammation in mice is still poorly understood. Oral treatment with ST-W77 and SE-W109 reduced inflammation caused by *S.* Tm infection by reducing proinflammatory response in the colon and spleen. This anti-inflammatory effect of both phages could result from the fewer numbers of invading *S.* Tm compared to the untreated mice.

Phages ST-W77 and SE-W109 reduced the shedding amount of *S.* Tm in mouse feces on days 1 and 4 but not on days 2 and 3 p.i (Figure 3). On day 1 p.i., we found that both phages significantly reduce the proliferation of *S.* Tm. Later, *S.* Tm numbers were increased on days 2, 3, and 4 p.i. probably due to the change in gut micro-environment from the induced inflammation that enhances *S.* Tm growth (Lupp et al., 2007). In the mouse colitis model of NTS, the most prominent inflammation occurs on day 4 p.i. and we found that both phages were significantly reduced *S.* Tm at this time point. Both phages can persist well in the gut lumen and tissue, including the mucus layer but not in the spleen (Figure 4). A previous study showed that phage adherence to the mucus layer increases the protection against pathogenic bacteria by providing phages a medium for interacting with invading bacteria (Almeida et al., 2019). Nonetheless, in this study, we did not quantify mucus-specific phage numbers.

Recently, there are growing pieces of evidence that support the benefit of using a mouse model to evaluate the efficacy and safety of *Salmonella* phage *in vivo* (Lamy-Besnier et al., 2021; Kumar et al., 2022). Here, we also showed that a mouse colitis model for acute NTS could be used to determine the efficacy of phages in reducing *Salmonella* numbers and *Salmonella*-induced inflammation.

## Conclusion

Therapeutic administration of *Salmonella* phages ST-W77 and SE-W109 can reduce *S.* Tm numbers and *S.* Tm-induced inflammation in both *in vitro* (human gut epithelium) and *in vivo* (mouse colitis model). ST-W77 and SE-W109 reduced proliferating and invading *S.* Tm subpopulations in a mammalian inflamed gut, resulting in decreased intestinal inflammation. A mouse model for acute human NTS should be a valuable tool to investigate the efficacy and safety of phage therapy in a pre-clinical setting.

## Data availability statement

The raw data supporting the conclusions of this article will be made available by the authors, without undue reservation.

## Ethics statement

The animal study was reviewed and approved by the Animal Care and Use Committee, Chiang Mai University, Thailand (Approval number 2563/MC-0002).

## Author contributions

PT and SK generated the research and critically reviewed the final version of manuscript. PT, SK, and CS designed and conceptualized the research. CS, SB, AT, and PT performed the research. TK performed the histopathological study. CS, SB, AT, SK, and PT analyzed the data. CS and PT drafted the manuscript. PP, JN, AL-G, MA, MFA, DM, EG, and MC supervised and edited the manuscript. All authors contributed to the article and approved the submitted version.

## Funding

This work was funded by Biotechnology and Biological Sciences Research Council (BBSRC), grant number RM38G0140 awarded to MC, and the National Science and Technology Development Agency (NSTDA), grant number P-18-50454 awarded to SK, the Faculty of Medicine Research Fund (MIC-2561-05411), Chiang Mai University, Chiang Mai, Thailand and the Thailand Research Fund (RGNS 63-066) awarded to PT. CS was awarded by the Faculty of Medicine Chiang Mai University graduate student scholarship. AT was awarded by the Chiang Mai University (CMU) Presidential scholarship.

## Supplementary material

SUPPLEMENTARY FIGURE S1 The schematic of phage treatment in *S.* Typhimurium-infected mouse model established in this study. After one-week acclimatization, 6–8 week-old female C57BL/6NJcl mice were orally fed with 100 μl of 200 mg/ml streptomycin sulfate 1 day before *S.* Typhimurium oral infection with 100 μl of 10^9^ CFU/ml (10^8^ CFU/mouse). Infected mice were divided into 4 groups (7 mice per group) and orally fed 100 μl of 10^11^ PFU/mL (10^10^ PFU/mouse) of ST-W77, SE-W109, ST-W77 combined with SE-W109, and SM buffer as the indicated time point. All mice were euthanized on day 4 post-infection.

SUPPLEMENTARY TABLE S1 The list of bacterial and phage strains used in this study.

SUPPLEMENTARY TABLE S2 The list of primers used in this study.

SUPPLEMENTARY TABLE S3 The criteria for mouse cecal histopathological scoring used in this study (Buddhasiri et al., 2021).
