# Supplementary material for: Therapeutic effects of oral administration of lytic Salmonella phages in a mouse model of non-typhoidal salmonellosis
Source: Front Microbiol. 2022 Oct 10;13:955136. doi: 10.3389/fmicb.2022.955136 (PMC9589268; doi:10.3389/fmicb.2022.955136)
Supplement: SUPPLEMENTARY FIGURE S1 — The schematic of phage treatment in S. Typhimurium-infected mouse model established in this study. After one-week acclimatization, 6–8 week-old female C57BL/6NJcl mice were orally fed with 100 μl of 200 mg/ml streptomycin sulfate 1 day before S. Typhimurium oral infection with 100 μl of 109 CFU/ml (108 CFU/mouse). Infected mice were divided into 4 groups (7 mice per group) and orally fed 100 μl of 1011 PFU/mL (1010 PFU/mouse) of ST-W77, SE-W109, ST-W77 combined with SE-W109, and SM buffer as the indicated time point. All mice were euthanized on day 4 post-infection. [file Table_1.pdf]

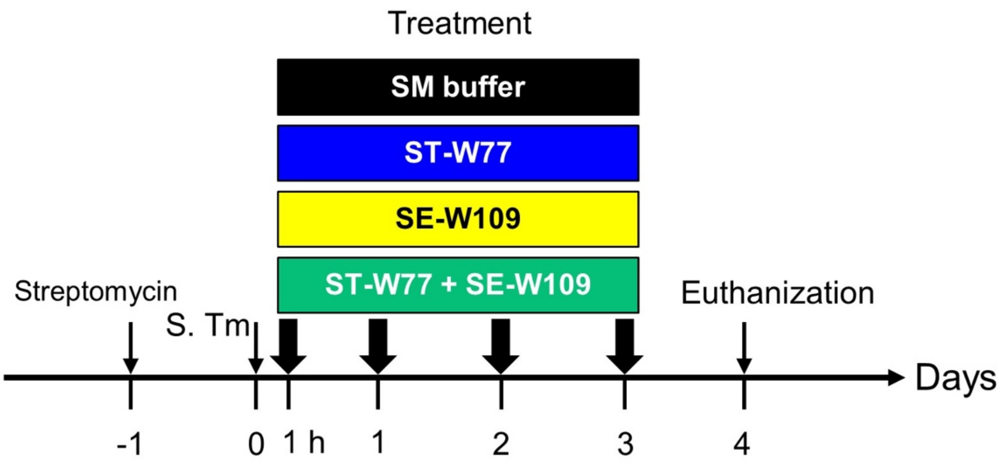

Figure S1: The schematic of phage treatment in *S.* Typhimurium-infected mouse model established in this study. After one-week acclimatization, 6-8 week-old female C57BL/6NJcl mice were orally fed with 100 µL of 200 mg/mL streptomycin sulfate one day before *S.* Typhimurium oral infection with 100 µL of 10^9 CFU/mL (10^8 CFU/mouse). Infected mice were divided into 4 groups (7 mice per group) and orally fed 100 µL of 10^{11} PFU/mL (10^{10} PFU/mouse) of ST-W77, SE-W109, ST-W77 combined with SE-W109, and SM buffer as the indicated time point. All mice were euthanized on day 4 post-infection.

Table S1. The list of bacterial and phage strains used in this study

Designation	Genotype and relevant characteristic	Reference
S. Typhimurium IR715 (*S.* Tm)	Nalidixic acid-resistant derivative of ATCC 14028	(1)
ST-W77	Myovirus within the Viunalikevirus	(2)
SE-W109	Siphovirus within the Jerseylikevirus	(2)

Table S2. The list of primers used in this study

Organism	Target gene	Primers	Reference
Homo sapiens	*GAPDH*	5’-CCAGGAAATGAGCTTGACAAAGT-3’<br>5’-CCCACTCCTCCACCTTTGAC-3’	(3)
	IL-8	5’-GCCAACACAGAAATTATTGTAAAGCTT-3’<br>5’-CCTCTGCACCCAGTTTTCCTT-3’	(4)
	MIP-3A	5’-CTGCTTTGATGTCAGTGCTGCTAC-3’<br>5’-CTGCCGTGTGAAGCCCACAATAAA-3’	(5)
	IL-1B	5’-AAACAGATGAAGTGCTCCTTCCAGG-3’<br>5’-TGGAGAACACCACTTGTTGCTCCA-3’	(6)
Mus musculus	*Gapdh*	5’-TGTAGACCATGTAGTTGAGGTCA-3’<br>5’-AGGTCGGTGTGAACGGATTTG-3’	(7)
	Kc	5’-TGCACCCAAACCGAAGTCAT-3’<br>5’-TTGTCAGAAGCCAGCGTTCAC-3’	
	Nos2	5’-CCAGCCTTGCATCCTCATTGG-3’<br>5’-CCAAACACCAAGCTCATGCGG-3’	
	IL-17	5’- GCTCCAGAAGGCCCTCAGA-3’<br>5’- AGCTTTCCCTCCGCATTGA-3’	(8)
	IFN-g	5’- TCAAGTGGCATAGATGTGGAAGAA-3’<br>5’- TGGCTCTGCAGGATTTTCATG-3’	(8)
	Zo-1	5’-CAGGGCTCTTTGGAGGAA-3’<br>5’-TACACGATCGTGGCAATAAAC-3’	(9)

Table S3. The criteria for mouse cecal histopathological scoring used in this study (10)

Score	Neutrophils infiltration	Mononuclear leukocyte infiltration	Submucosal edema	Epithelial damage	Exudate
0	No changes (0-5)	No changes (0-5)	No changes	No changes	No changes
1	6-20	6-10	Detectable (<10%)	Desquamation	Slight accumulation
2	21-60	11-20	Mild (10-20%)	Mild erosion and mild loss of goblet cells / undifferentiated enterocytes hyperplasia	Mild accumulation
3	61-100	21-40	Moderate (21-40%)	Marked erosion and moderate loss of goblet cells / undifferentiated hyperplasia	Moderate accumulation
4	>100	>40	Marked (>40%)	Ulceration and marked loss of goblet cells / undifferentiated hyperplasia	Marked accumulation

References:

1. Stojiljkovic I, Baumler AJ, Heffron F. Ethanolamine utilization in Salmonella typhimurium: nucleotide sequence, protein expression, and mutational analysis of the cchA cchB eutE eutJ eutG eutH gene cluster. J Bacteriol. 1995;177(5):1357-66.
2. Phothaworn P, Supokaivanich R, Lim J, Klumpp J, Imam M, Kutter E, et al. Development of a broad-spectrum Salmonella phage cocktail containing Viunalike and Jerseylike viruses isolated from Thailand. Food Microbiol. 2020;92:103586.
3. Raffatellu M, Chessa D, Wilson RP, Dusold R, Rubino S, Baumler AJ. The Vi capsular antigen of Salmonella enterica serotype Typhi reduces Toll-like receptor-dependent interleukin-8 expression in the intestinal mucosa. Infection and immunity. 2005;73(6):3367-74.
4. Stylianou E, Yndestad A, Sikkeland LI, Bjerkeli V, Damas JK, Haug T, et al. Effects of interferon-alpha on gene expression of chemokines and members of the tumour necrosis factor superfamily in HIV-infected patients. Clin Exp Immunol. 2002;130(2):279-85.
5. Johanesen PA, Dwinell MB. Flagellin-independent regulation of chemokine host defense in Campylobacter jejuni-infected intestinal epithelium. Infection and immunity. 2006;74(6):3437-47.
6. Jung HC, Eckmann L, Yang SK, Panja A, Fierer J, Morzycka-Wroblewska E, et al. A distinct array of proinflammatory cytokines is expressed in human colon epithelial cells in response to bacterial invasion. J Clin Invest. 1995;95(1):55-65.
7. Winter SE, Thiennimitr P, Winter MG, Butler BP, Huseby DL, Crawford RW, et al. Gut inflammation provides a respiratory electron acceptor for Salmonella. Nature. 2010;467(7314):426-9.
8. Winter SE, Thiennimitr P, Nuccio SP, Haneda T, Winter MG, Wilson RP, et al. Contribution of flagellin pattern recognition to intestinal inflammation during Salmonella enterica serotype typhimurium infection. Infection and immunity. 2009;77(5):1904-16.
9. Inagaki-Ohara K, Sawaguchi A, Suganuma T, Matsuzaki G, Nawa Y. Intraepithelial lymphocytes express junctional molecules in murine small intestine. Biochemical and biophysical research communications. 2005;331(4):977-83.
10. Buddhasiri S, Sukjoi C, Kaewsakhorn T, Nambunmee K, Nakphaichit M, Nitisinprasert S, et al. Anti-inflammatory Effect of Probiotic Limosilactobacillus reuteri KUB-AC5 Against Salmonella Infection in a Mouse Colitis Model. Front Microbiol. 2021;12:716761.
